# Apigenin supplementation substantially improves rooster sperm freezability and post-thaw function

**DOI:** 10.1038/s41598-024-55057-x

**Published:** 2024-02-24

**Authors:** Abouzar Najafi, Hossein Mohammadi, Seyed Davood Sharifi, Amin Rahimi

**Affiliations:** 1 Department of Animal and Poultry Science, Faculty of Agricultural Technology, College of Agriculture and Natural Resources, University of Tehran, Tehran, Iran; 2 Department of Animal Science, Faculty of Agriculture and Natural Resources, Arak University, Arak, Iran; 3 Chaltasian Agri.-Animal Production Complex, Varamin, Tehran Iran

**Keywords:** Rooster, Sperm, Antioxidant, Apigenin, Cryopreservation, Reproductive biology, Physiology

## Abstract

This pioneering research investigated apigenin potential to augment rooster sperm cryosurvival in an extender model. Apigenin is a natural antioxidant flavonoid showing promise for improved post-thaw sperm function. However, its effects on avian semen cryopreservation remain unexplored. This first study supplemented rooster sperm Lake extender with 0, 50, 100, 200, 400 μmol/L apigenin to determine the optimal concentrations for post-thaw quality. Supplementation with 100 μmol/L apigenin resulted in significant enhancements in total motility (from 41.5% up to 71.5%), progressive motility (18.1% to 29.1%) (p < 0.05), membrane integrity (40% to 68%), mitochondrial function (p < 0.001), viability (37% to 62%) and total antioxidant capacity (p < 0.001) compared to the control. It also substantially reduced percentages of abnormal morphology, reactive oxygen species and apoptosis (p < 0.001). Although 200 μmol/L apigenin significantly enhanced some attributes, effects were markedly lower than 100 μmol/L. Higher doses did not improve cryoprotective parameters. This indicates 100 μmol/L as the optimal apigenin concentration. This represents the first report of apigenin protecting rooster sperm from cryodamage. The natural antioxidant improved post-thaw sperm quality, likely by suppressing oxidative stress and apoptosis. Apigenin shows promise for enhancing rooster sperm cryosurvival.

## Introduction

Freezing semen is a good way to keep sperm cells alive in birds, but it can cause thermal shock to the sperm. Avian sperm cryopreservation poses unique challenges compared to other species. Avian sperm membranes contain high proportions of polyunsaturated fatty acids, making them vulnerable to freeze–thaw damage from reactive oxygen species (ROS)^1–3^. Lipid peroxidation impairs membrane integrity and function^4,5^. Rapid loss of internal ATP during cryopreservation may also irreversibly damage avian sperm^1^. Despite difficulties, successful cryopreservation protocols have been developed for valuable genetic lines of chickens, turkeys, ducks and geese using glycerol or dimethylacetamide (DMA) as permeating cryoprotectants^6–8^. Optimal cooling rates, straw sizes and thawing rates have been elucidated^4,8,9^. Non-permeating agents like liposomes, egg yolk or polymers provide additional sperm protection when added to standard glycerol or DMA freezing media^10–12^. Recently, supplementation with natural antioxidants has shown significant promise for improving post-thaw quality and fertility. Plant compounds such as crocin and quercetin^13,14^ or peptides like Type III antifreeze protein (AFP)^15^ have cryoprotective properties. They maintain sperm membrane integrity, motility and mitochondrial function by supressing ROS production and lipid peroxidation^13–15^. Apigenin is a plant-based compound that belongs to the flavonoid family. It has been studied for its possible benefits in preserving sperm quality during freezing and thawing processes. Some studies have shown that apigenin can improve the survival and function of sperm cells after cryopreservation, especially in bulls and boars^17,18^. For example, a study on bull semen found that adding 0.2 mmol/L of apigenin to the freezing solution improved the sperm ability to move, maintain their shape, and penetrate of the egg. Apigenin also increased the antioxidant activity of the sperm, which helped them resist against oxidative damage caused by freezing and thawing^17^. However, it is important to note that the concentration of apigenin used can have different effects. A study on mouse sperm production revealed that certain doses of apigenin interfered with the growth of sperm cells, which could lead to lower sperm count and higher abnormal sperm count^19^.

The possibility of improving the freezing survival of rooster sperm with a natural antioxidant such as apigenin is very attractive. For poultry breeders, frozen sperm allows easier global transport of elite livestock genetics to accelerate genetic gain^2^. Cryopreserved semen provides a practical way to rapidly disseminate desirable traits worldwide. Additionally, frozen sperm banks help ensure valuable poultry biodiversity is not lost, by preserving rare, threatened or unique avian genetic lines^1,6^. The techniques and specialized freezing extenders emerging from research can be implemented commercially to enhance production. Despite known difficulties freezing cockerel sperm compared to other species, elucidating treatments that boost cryosurvival rates will impact diverse fields spanning food production, conservation and genetics research on an international level^7,8^. This highlights the need to explore innovative freezing protocol modifications like antioxidant supplementation. There are many fruitful scientific areas and practical uses to explore if apigenin works well in this new bird model. While apigenin has shown great promise in protecting sperm from freeze–thaw injuries in bulls and boars^17,18^, its effects on avian semen cryopreservation remain unexplored. The aim of this study was to investigate the optimal dose of apigenin for enhancing the cryosurvival of rooster sperm after freezing and thawing. In pursuit of this goal, we conducted experiments testing different concentrations of apigenin (0, 50, 100, 200 and 400 μmol/L) supplemented into sperm Lake cryopreservation medium to determine the ideal level for preserving post-thaw quality.

## Materials and methods

### Chemicals and animal ethics

All chemical reagents were obtained from Sigma (St. Louis, MO), and Merck (Darmstadt, Germany). All animal care procedures were conducted in compliance with ARRIVE guidelines and the University of Tehran guidelines for Animal Experiments. The animal study was authorized by the Animal Research Committee of the University of Tehran.

### Animals used

The experiment was performed on 10 Cobb broiler breeder roosters (32 weeks old) housed in individual cages (70 × 95 × 85 cm) at 18 to 20 °C, when imposing a 15 L: 9 D photoperiod. Roosters were fed a commercial formula containing 12% crude protein and 2750 kcal/kg metabolizable energy, with 0.7% calcium and 0.35% available phosphorus. Water was provided ad libitum throughout the study period.

### Sperm collection

Semen was collected by abdominal massage eight times a month (twice a week). For quality evaluation, ejaculates from the ten roosters were taken to the laboratory within 5 min of collection in an insulated container at 37 °C. Samples meeting the following criteria were pooled to form each experimental unit: sperm volume (> 0.2 mL); sperm concentration (> 3 × 10^9^ sperm cells/ml) that was measured by a hemocytometer using a light microscope; total motility (> 80%) that was measured by a light microscope and normal morphology (85%) that was measured by the Hancock test. In total, eight pooled ejaculate samples were obtained, with each pool comprising equal semen aliquots from the ten study roosters. This approach aimed to minimize individual male variability when assessing semen cryopreservation responses^20^.

### Cryopreservation procedure

This study used a Lake-based extender^21^ buffer as the base freezing medium. The extender osmolality and pH were adjusted to 310 mOsm/kg and 7.1 respectively. Glycerol was then added to this basic medium at 3.8% (v/v) as the primary cryoprotectant. Apigenin was supplemented into the glycerol-containing Lake extender to achieve final treatment concentrations of 0, 50, 100, 200 and 400 μmol/L apigenin. Then, diluted semen was loaded into French straws (0.25 ml, IMV, L'Aigle, France) to obtain 100 × 10^6^ sperm cells/straws. The straws were closed with polyvinyl alcohol powder and then equilibrated at 4 °C for 3 h. Next, the straws were placed 6 cm above the liquid nitrogen surface for 10 min in a cryobox and then stored in a liquid nitrogen tank^22^. After storing for one week, straws were thawed at 37 °C for 30 s in a water bath and evaluated after thawing.

### Computer-assisted sperm analysis

A computer-assisted sperm analyzer (CASA V 12.2; Hamilton Thorne Biosciences, Beverly, MA, USA) was used to measure motion characteristics of sperm. For CASA analysis, thawed semen samples were diluted 1:10 with Lake buffer to obtain an optimal sperm concentration of 20 × 10^6^ sperm/mL. Three µL of thawed sperm were put on a prewarmed chamber (Leja, Nieuw-Vannep, Netherlands). The following CASA settings were used: frame rate 60 Hz; number of frames acquired 30; minimum contrast 60; particle area = 5 < 190 mm^2^. For each assessment, at least five fields were randomly scanned (200 cells minimum per evaluation) and then the following parameters were measured for the total motility (%), progressive motility (%), straightness (%), average path velocity (µm/s) straight line velocity (µm/s), curvilinear velocity (µm/s), amplitude of lateral head displacement (µm), beat cross-frequency (Hz) and linearity (%)^23^.

### Plasma membrane integrity

The sperm membranes evaluation was done by the hypo-osmotic swelling test (HOST)^21^. For this procedure, 10 μL of semen sample was combined with 100 μL of hypo-osmotic solution (9 g fructose + 4.9 g sodium citrate per liter of distilled water with an osmolality of 100 mOsm) and incubated at 37 °C for 30 min. Next, 5 μL of the mixture was placed on a slide that was pre-warmed and covered with a coverslip. A total of 200 sperm from at least ten microscopic fields was counted for the percentage of sperms with swollen and curled tails under a phase-contrast microscope (400× magnification).

### Abnormal morphology

To assess abnormal morphology, semen sample (10 µL) was mixed with Hancock’s solution (1 mL) that contained 65.5 mL formalin (37%), 150 mL normal saline solution, 150 mL buffer solution and, 500 µL double-distilled water. Then, 5 µL of the mixture was placed on a slide, covered with a coverslip. The proportion of sperm with abnormal acrosome, detached heads, abnormal mid-pieces and, tail were estimated by counting 300 sperm under a phase-contrast microscope^21^.

### TAC, SOD and GPx evaluation

The total antioxidant status (TAS) assay kit (NX2332) kit method (RANDOX Laboratories Ltd.) was used to measure total antioxidant capacity. This method is based on the scavenging of ABTS cation radical by antioxidants. The 20 μL of the samples were mixed with 1 ml of chromogen (ABTS reagent) and the reaction was triggered by adding 200 μL of H_2_O_2_. This method produced a stable blue-green color with a maximum light absorption of 600 nm, which can be measured by spectrophotometry. GPX enzyme activity was measured using RANSEL (SD125). In brief, 10 μL of sample was mixed with 500 µL of GPx reagent (glutathione, glutathione reductase and NADPH) and 10 µL of buffer (EDTA and phosphate buffer), then 4 µL cumene hydroperoxide was added to the mixture. The absorption was measured at a wavelength of 340 nm. RANSOD kit (SD125) was used to measure superoxide dismutase (SOD) activity. The assay is based on the generation of superoxide radicals produced by xanthine and xanthine oxidase, which react with INT to form a red formazan dye. SOD activity is then measured by the degree of inhibition of this reaction. Briefly, 50 μL of the sperm samples were mixed with 1.7 mL of mixed substrate solution containing xanthine and INT. Then, 500 μL of xanthine oxidase solution was added to start the reaction. The absorption was measured at a wavelength of 505 nm^14^.

### Assessment of ROS

The method of Mehdipour, et al.^21^ was used to measure ROS. The semen samples were incubated for 20 min in 250 mL of PBS at 37 °C and then centrifuged at 300*g* for 7 min, and the supernatant was removed. Then, 3 mL of PBS was added to the pellet and the mixture was centrifuged at 300*g* for 7 min. The sperm concentration was adjusted to 20 × 10^6^ mL by diluting the sperm with PBS. Next, 10 µL of luminol was added to 400 µL of sample and the tubes were placed in an Orion II Microplate Luminometer (Berthold Detection Systems GmbH, Pforzheim. Germany) for evaluation. The results were expressed as 10^3^ cpm/10^6^ spermatozoa.

### Flow cytometry analysis

A FACSCalibur flow cytometer (Becton Dickinson) equipped with a 488-nm argon laser was employed for assessments of phosphatidylserine externalization and mitochondrial activity**.** The system contained 530/30 nm (Annexin V-FITC and rhodamine 123) and 610/20 nm (PI) bandpass filters for detection of green and red fluorescence emission, respectively. In all analyses, sperm were gated based on forward/side scatter signals and 10,000 cells acquired per sample. Quantitative flow cytometry data were recorded using CellQuest 3.3 software.

### Phosphatidylserine externalization

The externalization of phosphatidyl serine as an indicator of apoptosis in sperm cells was evaluated by an Annexin V-FITC kit (IQP, Groningen, and The Netherlands) and PI. The samples were rinsed in calcium buffer and adjusted to the concentration of 1 × 10^6^ spermatozoa/ml. Then, 5 µL Annexin V FITC (0.01 mg/ml) was added to 100 µL of the sperm suspension and incubated for 20 min at room temperature (22 °C). Next, 5 µL of PI were added to the sperm suspension and incubated for at least 10 min at 22 °C. The sperm suspension was analyzed by flow cytometry after Annexin V-FITC and PI staining, and sperm subpopulations were divided into four groups^24^: (1) viable sperm exhibiting an Annexin V-FITC negative and PI negative; (2) early apoptotic sperm displaying Annexin V-FITC positive and PI negative staining; (3) late apoptotic sperm with an Annexin V-FITC positive and PI positive; and (4) necrotic sperm showcasing Annexin V-FITC negative and PI positive staining. The late apoptotic and necrotic subgroups exhibiting PI uptake were presumably classified as dead sperm^21^.

### Mitochondrial activity

To assess the mitochondrial activity of sperm, diluted semen samples (100 mL; 50 × 10^6^ sperm/mL) were mixed with 5 µL of Rhodamine 123 (R123; Invitrogen, Eugene, OR) (0.01 mg/mL) and kept in the dark at room temperature. Next, 5 µL of PI solution (1 mg/mL) was added and the sample was analyzed by flow cytometry to determine the total number of live sperm with active mitochondria (R123 positive and PI negative)^23^.

### Statistical analysis

Pooled semen was cryopreserved using eight replicates and sperm evaluation was done as described earlier in this manuscript. Data were analyzed by linear mixed-effects models using the R statistical system (R Team, 2023). The results are shown as mean ± SEM. Mean differences were considered when P ≤ 0.05, using Tukey’s method for multiple-comparison corrections.

## Results

**Figure 1:**
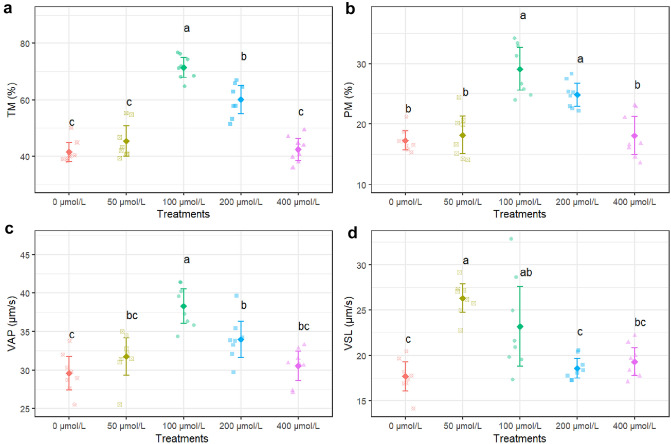
*TM* total motility (**a**), *PM* progressive motility (**b**), *VAP* average path velocity (**c**) and *VSL* straight-line velocity (**d**) after thawing samples cryopreserved with different apigenin concentrations. Different letters indicate that treatments differ P < 0.05.

**Figure 2:**
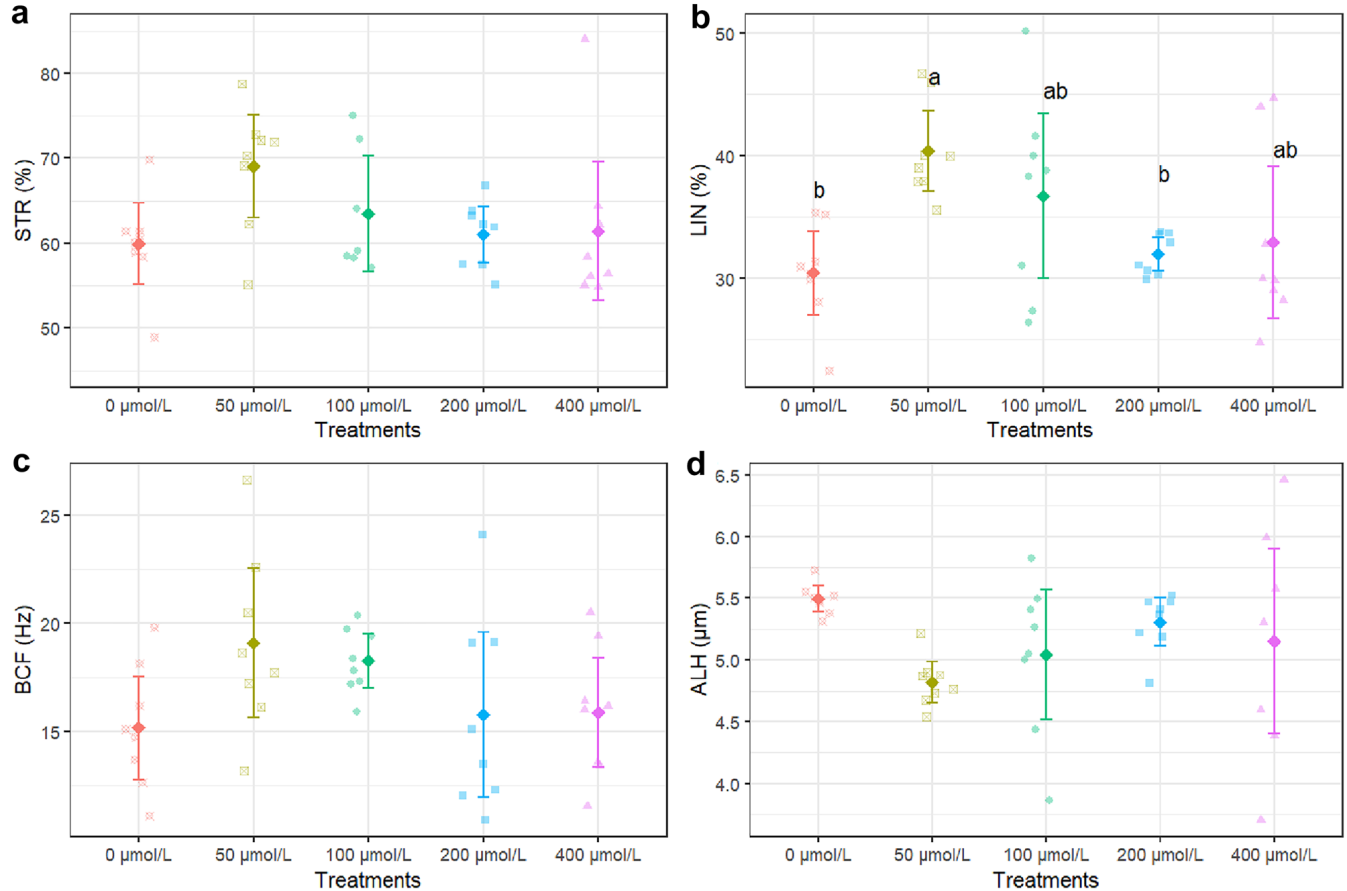
*STR* straightness (**a**), *LIN* linearity (**b**), *ALH* amplitude of the lateral head displacement (**c**) and *BCF* beat cross frequency (**d**) after thawing samples cryopreserved with different apigenin concentrations. Different letters indicate that treatments differ P < 0.05.

The result showed that 100 µmol/L apigenin significantly improved TM compared to all other treatments (P < 0.05), increasing it to 71.5% from 41.5% in the untreated control (Fig. 1a). Also 200 µmol/L also increased TM but was only significantly higher than the control and 400 µmol/L (Fig. 1a). Our result revealed that 100 µmol/L apigenin significantly enhanced PM to 29.1% compared to 18.1–17.3% in the other groups except 200 µmol/L which was not significantly different (Fig. 1b). Supplementation with 100 µmol/L apigenin increased VAP to 38.3 μm/s, which was significantly faster than all other doses based (Fig. 1c). Similar significant effects of 100 µmol/L apigenin were observed for other motility parameters like straight line velocity (VSL), and linearity (LIN) based on multiple comparisons (Figs. 1d and 2b). However, no treatment significantly altered straightness (STR), beat cross frequency (BCF) and amplitude of lateral head displacement (ALH) (Fig. 2a, c, d).

**Figure 3:**
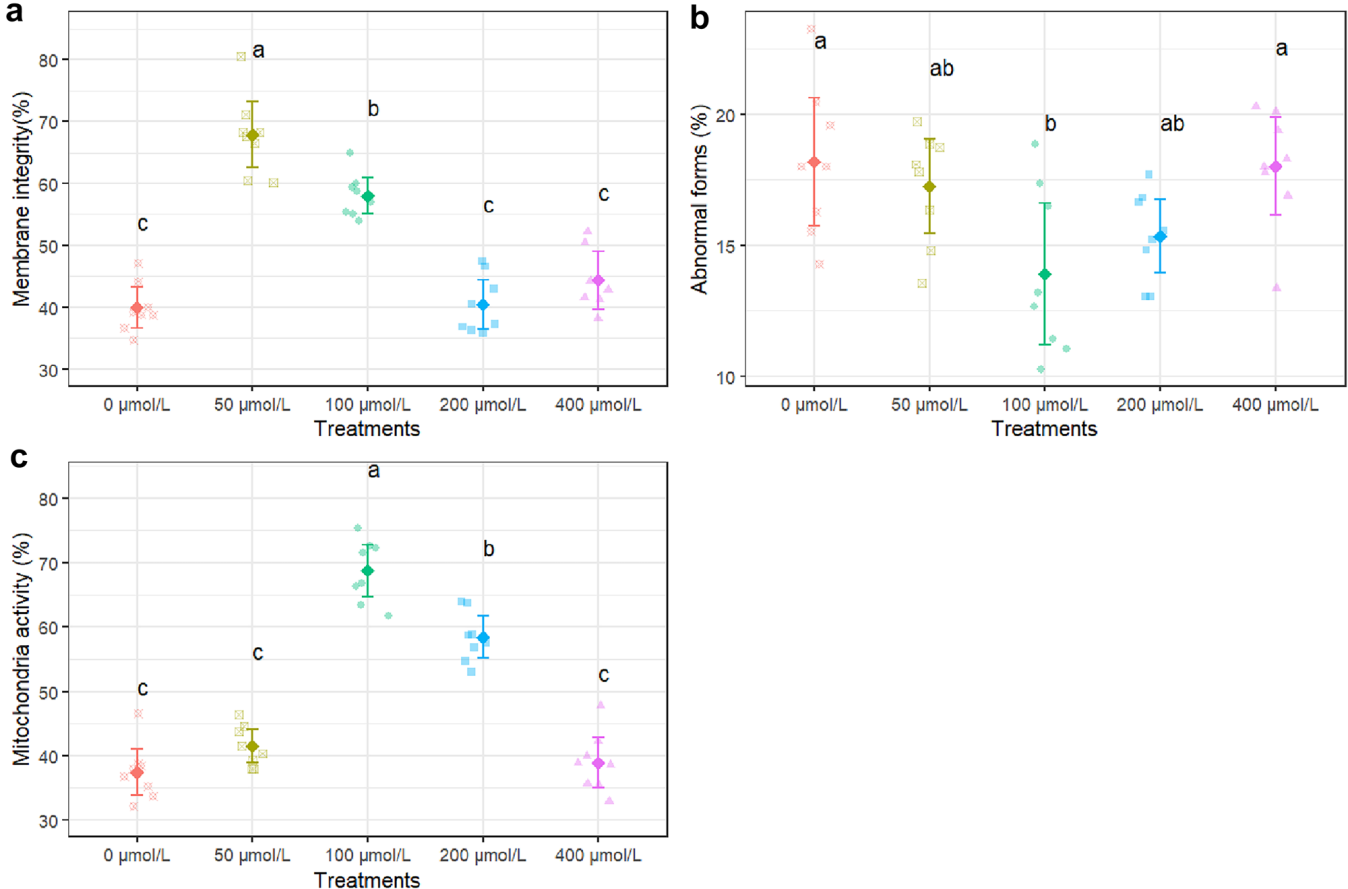
Membrane integrity (**a**), total abnormality (**b**), and mitochondrial activity (**c**) after thawing samples cryopreserved with different apigenin concentrations. Different letters indicate that treatments differ P < 0.05.

The 100 µmol/L apigenin treatment led to a substantial and highly significant (P < 0.001) increase in sperm membrane integrity compared to control, improving from 40% integrity in control to 68% integrity with 100 µmol/L apigenin (Fig. 3a). The 200 µmol/L apigenin dose also significantly enhanced sperm membrane integrity versus control (P < 0.001), elevating it from 40 to 59%. However, the improvement was less prominent compared to the effects of 100 µmol/L apigenin. The 100 µmol/L apigenin treatment led to a significant reduction (P < 0.05) in the percentage of sperm with abnormal forms compared to control, decreasing it from 18.2% in control to 13.9% with supplementation (Fig. 3b). Mitochondrial activity was significantly higher at 100 µmol/L compared to other concentrations (0, 50, 200, 400 µmol/L) (Fig. 3c).

**Figure 4:**
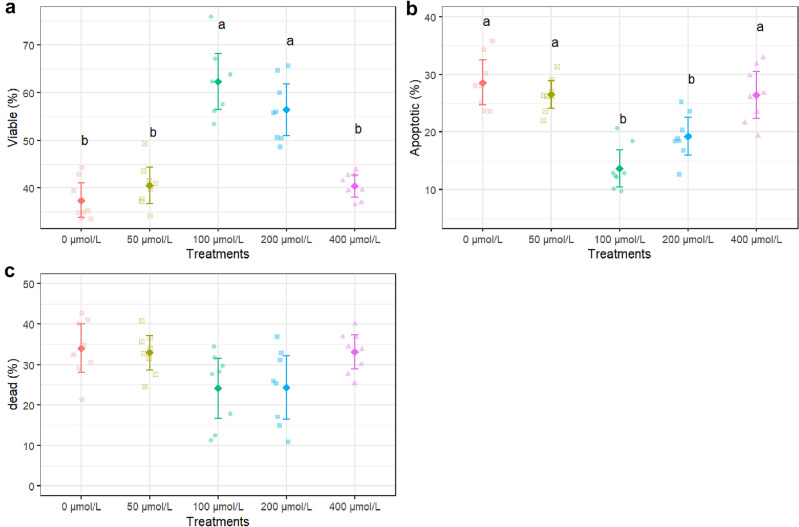
Viability (**a**), apoptotic (**b**) and dead sperm (**c**) after thawing samples cryopreserved with different apigenin concentrations. Different letters indicate that treatments differ P < 0.05.

Percentage of viable sperm improved remarkably with 100 µmol/L apigenin supplementation over control (P < 0.001), escalating from 37 to 62%. The 200 µmol/L apigenin dose also raised viable percentage significantly vs control (P < 0.001), up to 56% from 37% (Fig. 4a). However, the effect was lower than 100 µmol/L. Whereas on sperm apoptosis, 100 µmol/L and 200 µmol/L apigenin led to substantial and significant reductions (P < 0.001) compared to control (Fig. 4b). There were no significant differences between any groups in percentages of dead sperm (Fig. 4c).

**Figure 5:**
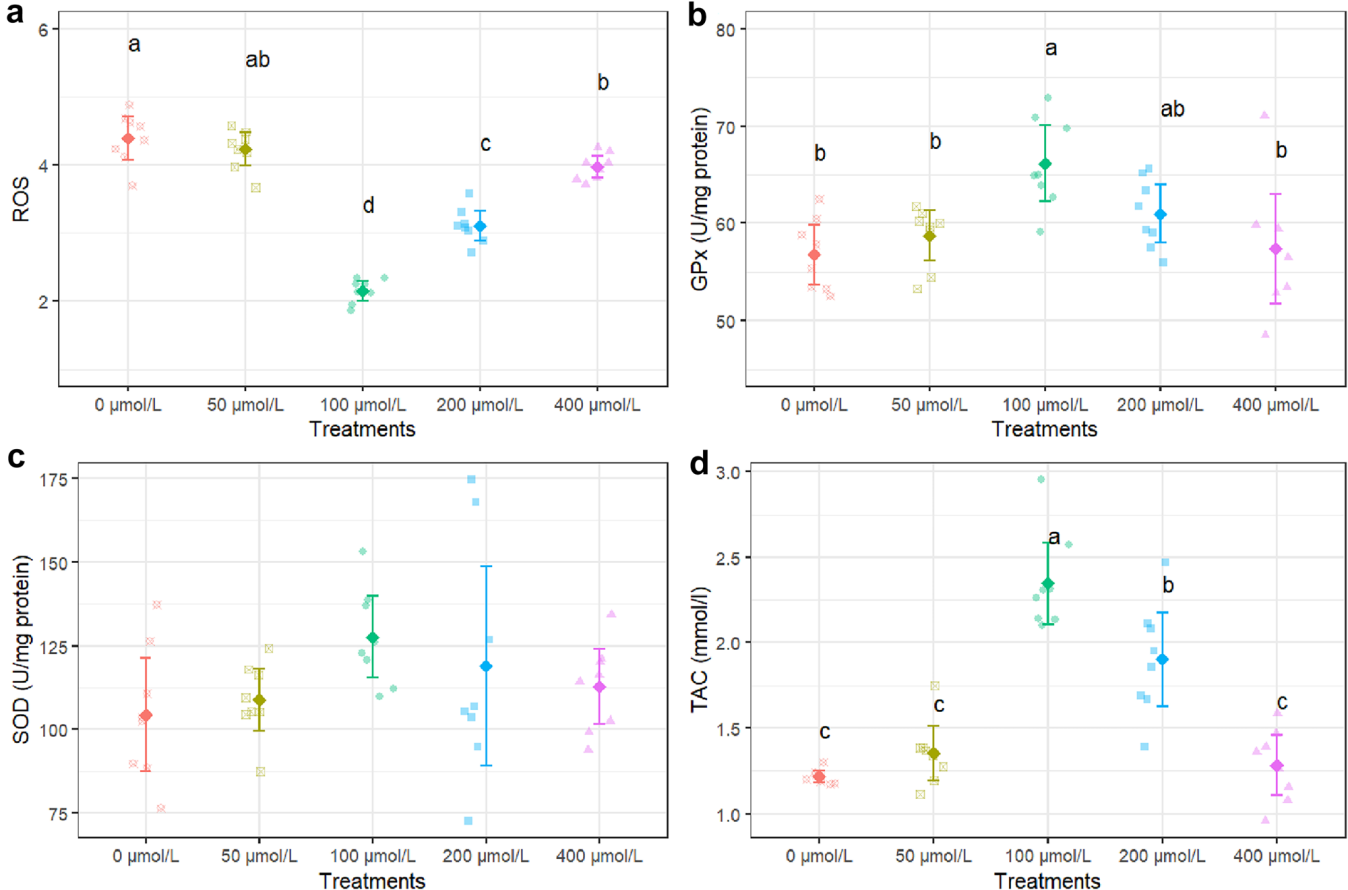
ROS (**a**), GPx (**b**), SOD (**c**), and TAC (**d**) after thawing samples cryopreserved with different apigenin concentrations. Different letters indicate that treatments differ P < 0.05.

Apigenin supplementation at doses of 100 µmol/L and 200 µmol/L led to significant reductions in sperm ROS levels compared to control (P < 0.001) (Fig. 5a). Apigenin supplementation did not cause significant improvements in sperm SOD levels compared to control across the doses tested (Fig. 5c). However, 100 µmol/L apigenin treatment markedly and significantly increased sperm TAC levels (P < 0.001). The 200 µmol/L dose also enhanced TAC significantly by 55% versus control (P < 0.001), although the effect was lower than 100 µmol/L (Fig. 5d). For GPX, only the 100 µmol/L apigenin dose led to a significant elevation versus control (P < 0.01) (Fig. 5b).

## Discussion

The aim of this study was to investigate the optimal dose of apigenin for enhancing the cryosurvival of rooster sperm. We found that 100 µmol/L apigenin significantly improved the total motility, progressive motility (PM), and velocity parameters (VAP, VSL, LIN, and STR) of frozen-thawed rooster sperm compared to the control and other doses. Interestingly the beneficial effects of apigenin were dose-dependent, with optimal enhancements observed at 100 µmol/L across parameters. The 200 µmol/L dose elevated motility, viability and integrity but the effects were smaller than 100 µmol/L, while 50 and 400 µmol/L did not impact parameters substantially. Apigenin has antioxidant properties that reduce oxidative stress on sperm at optimal low doses. The 100 µmol/L dose provided the most protection against ROS. This led to improved sperm quality parameters like motility.

At the optimal dose of 100 µmol/L, apigenin may activate MAPK and PKA pathways involved in motility via redox signaling^25,26^. This leads to precisely controlled phosphorylation of proteins such as axonemal components that regulate beating^27,28^. Apigenin may protect sperm membrane from oxidative damage and improve its fluidity and stability, thereby increasing the sperm viability and integrity^29,30^. This effect may be more pronounced at 100 µmol/L than at 200 µmol/L. Dang, et al.^31^, showed that apigenin could protect the mitochondrial midpiece and the outer dense fibers of the flagellum from damage in rat epididymal spermatozoa exposed to acrylonitrile. This could increase the sperm motility, which is crucial for successful fertilization. Wang, et al.^17^, investigated the impact of apigenin on the cryopreservation of bovine semen. The study found that addition of 0.2 mmol/L apigenin improved the quality of frozen sperm. Inconsistent of our result, Haleagrahara, et al.^32^, examined the protective effects of apigenin against cyclosporine-induced nephrotoxicity in rats. The study used three doses of apigenin (10, 15, and 20 mg/kg) in addition to cyclosporine treatment. The results showed that apigenin treatment significantly reduced the pathological changes caused by cyclosporine in the kidney. The protective effects of apigenin were attributed to its antioxidant properties, which helped to scavenge free radicals and prevent oxidative damage to the kidney. However, the study did not provide a specific recommended dosage of apigenin for improving sperm quality in rats.

It has been reported that antioxidants provide better protection to semen. It has been demonstrated that semen with sufficient antioxidant levels can resist freeze–thaw stress better^16,33^. Our results showed that apigenin modulated the antioxidant defense system in sperm, by reducing the ROS levels and increasing the TAC and GPX levels. This is in line with a study that demonstrated the antioxidant properties of apigenin on sperm and testis^34^. Apigenin may reduce oxidative stress and lipid peroxidation in sperm, by scavenging ROS and enhancing the activity of antioxidant enzymes, such as superoxide dismutase (SOD), glutathione peroxidase (GPX), and total antioxidant capacity (TAC)^34–36^. Kopalli, et al.^37^ investigated the protective effects of apigenin against oxidative damage in testicular sperm cells. The study demonstrated that apigenin might be an effective agent in protecting against ROS induced testicular dysfunctions. Also, Dang, et al.^31^, found that apigenin supplementation significantly improved sperm quality and reduced oxidative stress in rats exposed to acrylonitrile. The protective effects of apigenin were attributed to its antioxidant properties, which was effective in scavenging free radicals and prevent oxidative damage to sperm.

Apigenin may reduce apoptosis in sperm through antioxidant effects as well as direct anti-apoptotic mechanisms. The 100 µmol/L dose provided the most protection against apoptosis-induced DNA damage and loss of motility/function. Apigenin may inhibit sperm apoptosis and necrosis, by regulating the balance between pro-apoptotic and anti-apoptotic factors, such as caspases, Bcl-2, and Bax^31,34,35^. Shi, et al.^35^, investigated the protective effects of apigenin against acrylonitrile-induced sperm and testis injury in rats. The results suggested that apigenin treatment significantly improved sperm quality and reduced testicular injury in rats exposed to acrylonitrile. The protective effects of apigenin were attributed to its ability to activate the ASK1-JNK/p38 signaling pathway, which plays a role in regulating cell survival and apoptosis in response to oxidative stress. However, Wang, et al.^38^, reported that high dose apigenin resulted in reduced spermatogonia proliferation and increased abnormal sperm count in mice. Based on this study, it can be hypothesized that apigenin had pro-oxidant effects on sperm and testis at high doses, by increasing the ROS levels and decreasing the antioxidant enzyme activities, suggesting that the antioxidant or pro-oxidant effects of apigenin may depend on the dose and the duration of exposure.

Cryopreservation can impair the mitochondrial function of sperm and measuring the mitochondrial function is essential to evaluate the effectiveness of antioxidants for freezing methods^39^. Our results indicated that apigenin preserved better mitochondrial activity after thawing, which is consistent with other observable parameters such as motility, membrane integrity and oxidative status. Apigenin may increase the mitochondrial activity and energy production in sperm, which may be essential for sperm motility and function. This effect may be specific to 100 µmol/L, as the other doses did not show any significant difference from the control. The antioxidant properties of 100 µmol/L apigenin may inhibit lipid peroxidation of sperm plasma membranes and mitochondria. This preserves membrane fluidity and integrity by preventing the formation of reactive lipid aldehydes and loss of polyunsaturated fatty acids from phospholipid bilayers^18,31,40^. Critical membrane proteins involved in motility, capacitation and signal transduction are thus able to retain normal function^41^. In agreement to our result, Pei, et al.^18^, aimed to evaluate the cryoprotective effect of apigenin and ferulic acid (FA) on boar sperm during cryopreservation. The results suggested that both apigenin and ferulic acid significantly improved mitochondrial activity of the frozen-thawed boar sperm. The highest improvement was recorded when the extender was supplemented with 0.1 mmol/L apigenin plus 0.15 mmol/L ferulic acid.

## Conclusion

This study demonstrates that supplementation with 100 µmol/L apigenin significantly improves rooster sperm quality after cryopreservation across key parameters including motility, viability, integrity and antioxidant defenses. Importantly, a clear dose-dependent effect of apigenin concentration was observed, with optimal enhancements seen specifically at 100 μmol/L across both sperm quality attributes and functional biomarkers. By elucidating the precise apigenin dose for maximizing rooster sperm freezability, this research paves the way for better preservation of invaluable genetic lines to support sustainable poultry production into the future (Supplementary Information).

### Supplementary Information

Supplementary Information.

## Data Availability

The authors declare that the data supporting the findings of this study are available within the paper.
